# Consolidation treatment of durvalumab after chemoradiation in real‐world patients with stage III unresectable non‐small cell lung cancer

**DOI:** 10.1111/1759-7714.13426

**Published:** 2020-04-13

**Authors:** Chia‐Hsun Chu, Tzu‐Hsuan Chiu, Chin‐Chou Wang, Wen‐Chen Chang, Allen Chung‐Cheng Huang, Chien‐Ying Liu, Chih‐Liang Wang, Ho‐Wen Ko, Fu‐Tsai Chung, Ping‐Chih Hsu, Yi‐Ke Guo, Chih‐Hsi S. Kuo, Cheng‐Ta Yang

**Affiliations:** ^1^ Division of Thoracic Oncology, Department of Thoracic Medicine Chang Gung Memorial Hospital, Chang Gung University, College of Medicine Taoyuan City Taiwan; ^2^ Thoracic Oncology Unit Chang Gung Memorial Hospital Cancer Center Taoyuan City Taiwan; ^3^ Division of Pulmonary & Critical Care Medicine Kaohsiung Chang Gung Memorial Hospital Kaohsiung Taiwan; ^4^ Department of Medical Oncology Chang Gung Memorial Hospital, Chang Gung University Taoyuan City Taiwan; ^5^ Data Science Institute, Department of Computing Imperial College London London UK

**Keywords:** Chemoradiation, consolidation, durvalumab

## Abstract

**Background:**

Treatment for stage III non‐small cell lung cancer (NSCLC) of unresectable disease mainly involves concurrent chemoradiation (CRT). Post‐CRT consolidation treatment with durvalumab is a major therapeutic advance that provides survival benefit in this group of patients. However, the performance of this treatment strategy remains to be studied in a real‐world setting.

**Methods:**

A total of 31 patients who had disease control post‐CRT were included in the durvalumab early access program (EAP) as an intent‐to‐treat cohort and retrospectively reviewed for post‐CRT progression‐free survival (PFS) and time to metastatic disease or death (TMDD). The neutrophil‐to‐lymphocyte ratio (NLR) at the initiation of durvalumab was analyzed in 29 patients.

**Results:**

The median time from the completion of concurrent CRT to the initiation of durvalumb was 2.8 months. The objective response was 25.8% and the 12 month PFS and TMDD‐free rate were 56.4% and 66.9%, respectively. The low NLR patients showed a significantly longer post‐CRT PFS (not reach vs. 12.0 months [95% CI: 5.5–not estimable]; *P* = 0.040; the hazard ratio for disease progression or death, 0.23 [95% CI: 0.05–1.00]; *P* = 0.048) and the 12 month post‐CRT PFS rate (82.5 vs. 42.6%). The post‐CRT TMDD (not reach vs. 12.6 months, [95% CI: 10.8–not estimable]; *P* = 0.010; the hazard ratio for distant metastasis or death, 0.11 [95% CI: 0.01–0.88]; *P* = 0.037) and 12 month post‐CRT TMDD‐free rate (90.9 vs. 57.1%) were also significantly higher in the low NLR patients.

**Conclusions:**

Durvalumab consolidation treatment in real‐world patients showed substantial efficacy and the correlation with the NLR level warrants further investigation.

## Introduction

Stage III NSCLC represents a heterogeneous group of disease entities that are potentially curable and are usually dealt with multimodality treatments involving radiotherapy, chemotherapy, and surgical resection.^1^, ^2^ For patients with unresectable stage III disease, definitive chemoradiation delivered either concomitantly or sequentially has long been the standard of care whereas the survival rate beyond five years remains dismal at around 15%–30%.^3^, ^4^, ^5^

The poor long‐term survival for unresectable stage III NSCLC patients, as a result of the subsequent progression and metastasis of the residual disease following definitive chemoradiation, has been a major challenge that demands an effective consolidation treatment.^6^ Previous trials which studied the role of consolidation chemotherapy have mainly yielded disappointing results.^7^, ^8^, ^9^ Ahn *et al*. investigated a combination of docetaxel and cisplatin in a randomized phase III trial where it showed that the chemotherapy group using this combination was not superior to the control group in which the patients only received best supportive care after chemoradiation.^7^ The other phase III trial of similar study design, while applying a different chemotherapeutic combination, viorelbine plus cisplatin, also yielded a similar outcome.^8^ The lack of clinical benefit of consolidation chemotherapy was also noted in a meta‐analysis involving more than 3000 unresectable stage III NSCLC patients.^10^

One of the major reasons that patients failed to benefit from the consolidation chemotherapy could be blamed on the tolerance to this approach in the wake of definitive chemoradiation. In the earlier phase III study, a significant portion of the intent‐to‐treat population randomized to the consolidation chemotherapeutic arm was unable to initiate the treatment; moreover, nearly 40% of patients who initiated the treatment failed to follow the defined treatment protocol.^7^ In addition, one study that applied etoposide and cisplatin‐based chemoradiation noted that the subsequent consolidation chemotherapy was even associated with an increased treatment‐related infection and death.^9^

Recently, the strategy to apply immune checkpoint inhibitors as a consolidation treatment after chemoradiation has demonstrated promising results.^11^, ^12^ Previous studies have reported that chemoradiation can give rise to a number of immune reactions crucial to tumor containment. These include increased type I interferon and major histocompatibility complex (MHC) class I expression, as well as enhanced priming capacity of tumor‐infiltrating dendritic cells^13^, ^14^, ^15^; all of which may contribute to the increased tumor infiltration of the effector CD8 T cells.^16^, ^17^, ^18^ Given that, the subsequent administration of the programmed cell death 1 (PD‐1) or PD‐1 ligand (PD‐L1) inhibitors, acting to mitigate the PD‐1/PD‐L1‐mediated immunosuppression, sustains the effector immunity established post‐chemoradiation around the tumor microenvironment and thereby contain the residual disease through an operational immune surveillance.

In this regard, the PD‐L1 inhibitor durvalumab given in the wake of concurrent chemoradiation represented a major leap toward the consolidation strategies. In the phase III PACIFIC study,^11^, ^12^ durvalumab administered at six weeks post chemoradiation, compared to the placebo, showed a significantly longer PFS, time to distant metastasis and overall survival (OS). Another phase II study with a similar strategy applied the regimen of PD‐1 inhibitor pembrolizumab and has also reported a longer time to distant metastasis compared to the historical controls.^19^ Nevertheless, earlier studies have shown that radiotherapy may give rise to systemic lymphopenia^20^ and whether the level of peripheral white blood cells is associated with the efficacy of consolidation treatment using checkpoint inhibitors remains unclear. On the other hand, in a real world setting, the compliance and tolerance of consolidation treatment is often limited by the toxicities directly related to the definitive chemoradiation given ahead. As such, whether durvalumab consolidation in a real‐world setting has an equivalent performance as previous trials requires further investigation.

In the present study, we analyzed a group of stage III unresectable NSCLC patients who had disease control after concurrent chemoradiation and intended to receive consolidation treatment using durvalumab. We report herein the preliminary results of durvalumab consolidation in this group of intent‐to‐treat population.

## Methods

### Study patients

**Figure 1:**
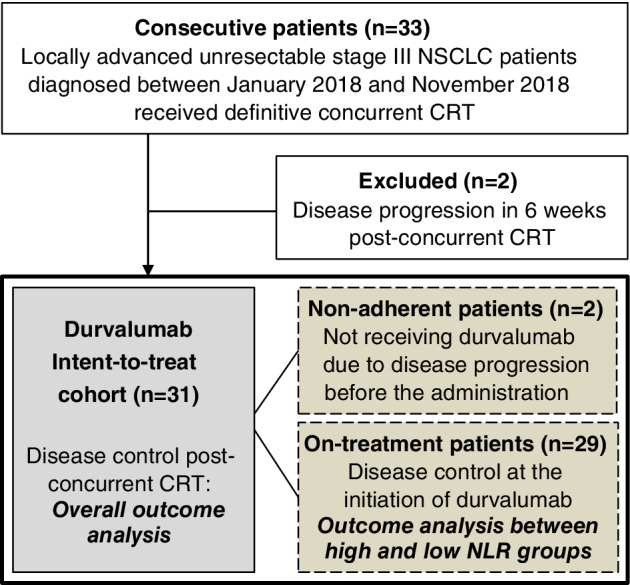
Flow chart of the study population in which the durvalumab intent‐to‐treat cohort and the on‐treatment patients received the major analysis. NLR, neutrophil‐to‐lymphocyte ratio.

Between January 2018 and November 2018, 33 consecutive patients (Fig 1) with histologically documented locally advanced unresectable stage III NSCLC based on chest computed tomography (CT) scan, magnetic resonance imaging, positron emission tomography (PET) and dedicated multidisciplinary assessment were retrospectively reviewed. Patients received concurrent chemoradiation therapy (CRT) with the protocol of radiotherapy 66–70 Gy in 32–35 fractions and chemotherapy using weekly docetaxel (20 mg/kg) or vinorelbine (15 mg/kg) plus cisplatin (20 mg/kg) for five weeks. A total of 31 (93.9%, Fig 1) who had disease control after concurrent CRT were entered into the durvalumab early access program (EAP), receiving consolidation treatment using durvalumab 10 mg/kg every two weeks, and were included in the overall efficacy analysis as the intent‐to‐treat cohort. Among these 31 patients, two patients underwent disease progression before the initiation of durvalumab and did not adhere to the treatment. PD‐L1 assessment was studied with a Dako PharmaDx 22C3 immunohistochemistry assay, and the tumor proportion score was calculated and reported as previously described.^21^ The study took advantage of the Chang Gung Research Database and was approved by the Ethics Committee of Chang Gung Memorial Hospital. The study was performed in accordance with the ethical standards of the 1964 Declaration of Helsinki. Written inform consents were provided by all study participants.

### Outcome assessment

After the completion of concurrent CRT, a CT scan was performed at six weeks and taken as the baseline image. All patients were confirmed with nonprogressive disease at the baseline assessment and received subsequent image studies every eight weeks. The overall post‐CRT PFS, calculated between the date of the radiological confirmation of disease control post‐CRT and the date of radiologically documented progression to durvalumab consolidation or death, was analyzed according to the intent‐to‐treat principle. A specific pattern of progression, the post‐CRT TMDD, was also analyzed according to the same principle. The treatment response, defined as complete response (CR), partial response (PR), stable disease (SD), and progressive disease (PD), was evaluated according to the Response Evaluation Criteria in Solid Tumors (RECIST) version 1.1. The toxicities of durvalumab were regularly assessed and recorded during the treatment course by the attending physician and the toxicity was graded according to the National Cancer Institute Common Toxicity Criteria, version 5.0.

### Statistical analysis

The Mann‐Whitney test was used to determine the statistical significance between two groups of continuous variables, and the Fisher's exact test was used for categorical variables. The Kaplan‐Meier survival curve was analyzed by the R package survival. The Cutoff Finder, an R language‐based web interface, was used to determine the cut points of the continuous variable.^22^ All reported *P*‐values were two sided, and a *P*‐value < 0.05 was considered statistically significant. All the data were also analyzed by SPSS v.25 (SPSS Corp., Chicago, IL, USA).

## Results

### Baseline patient characteristics

Among the 31 intent‐to‐treat patients (Table 1), 26 (83.9%) were male, 23 (74.2%) were smokers or ex‐smokers and 25 (80.7%) were in ECOG PS 0 when they received durvalumab treatment. A total of 20 patients (63.9%) had adenocarcinoma, eight (25.8%) had squamous cell carcinoma and three (9.7%) had NSCLC not otherwise specified (NOS). A total of 14 patients (45.2%) had a positive PD‐L1 expression and the frequency of *EGFR* and *ALK* driver mutations were noted in four (12.9%) and one (3.3%) patients, respectively. For the concurrent CRT protocol: 20 patients (63.9%) received a radiotherapy dose at 60–66 Gy, eight (25.8%) patients had a radiotherapy dose >66 Gy and three (9.7%) received the radiotherapy dose <60 Gy; docetaxel and cisplatin were used in 13 (41.9%) patients and vinorelbine and cisplatin were given in 18 (58.1%) patients. The median time for the initiation of durvalumb treatment after the completion of concurrent CRT was 2.8 (1.8–3.7) months.

**Table 1:** Clinical characteristics of all study subjects

Variable, n (%)	
Age, median (year)	64 (52–74)
Gender	
Male	26 (83.9)
Female	5 (16.1)
Smoking status	
Smoker/ex‐smoker	23 (74.2)
Never smoker	8 (25.8)
ECOG PS	
0	25 (80.7)
1	5 (16.1)
2	1 (3.2)
Histology	
Adenocarcinoma	20 (64.5)
Squamous cell carcinoma	8 (25.8)
NSCLC NOS	3 (9.7)
Staging	
IIIA	8 (25.8)
IIIB	21 (67.7)
IIIC	2 (6.5)
*EGFR* mutation status	
Mutated *EGFR*	4 (12.9)
Wild type	19 (61.3)
Unknown	8 (25.8)
*ALK* fusion status	
Positive	1 (3.3)
Negative	17 (54.8)
Unknown	13 (41.9)
PD‐L1 TPS	
Positive (≥1%)	14 (45.2)
Negative (<1%)	6 (19.3)
Unknown	11 (35.5)
Chemotherapy regimen	
Docetaxel plus cisplatin	13 (41.9)
Vinorelbine plus cisplatin	18 (58.1)
Dose of radiotherapy	
60–66 Gy	23 (74.2)
>66 Gy	8 (25.8)
Timing of durvalumab initiation post‐CCRT, median (month)	2.8 (1.8–3.7)
Total	31 (100.0)

ALK, anaplastic lymphoma kinase; CCRT, concurrent chemoradiation; ECOG PS, Eastern Cooperative Oncology Group performance status; EGFR, epidermal growth factor receptor; NOS, not otherwise specified; PD‐L1, Programmed death‐ligand 1; TPS, tumor proportion score.

### Outcome of post‐CRT tumor control with durvalumab consolidation

**Figure 2:**
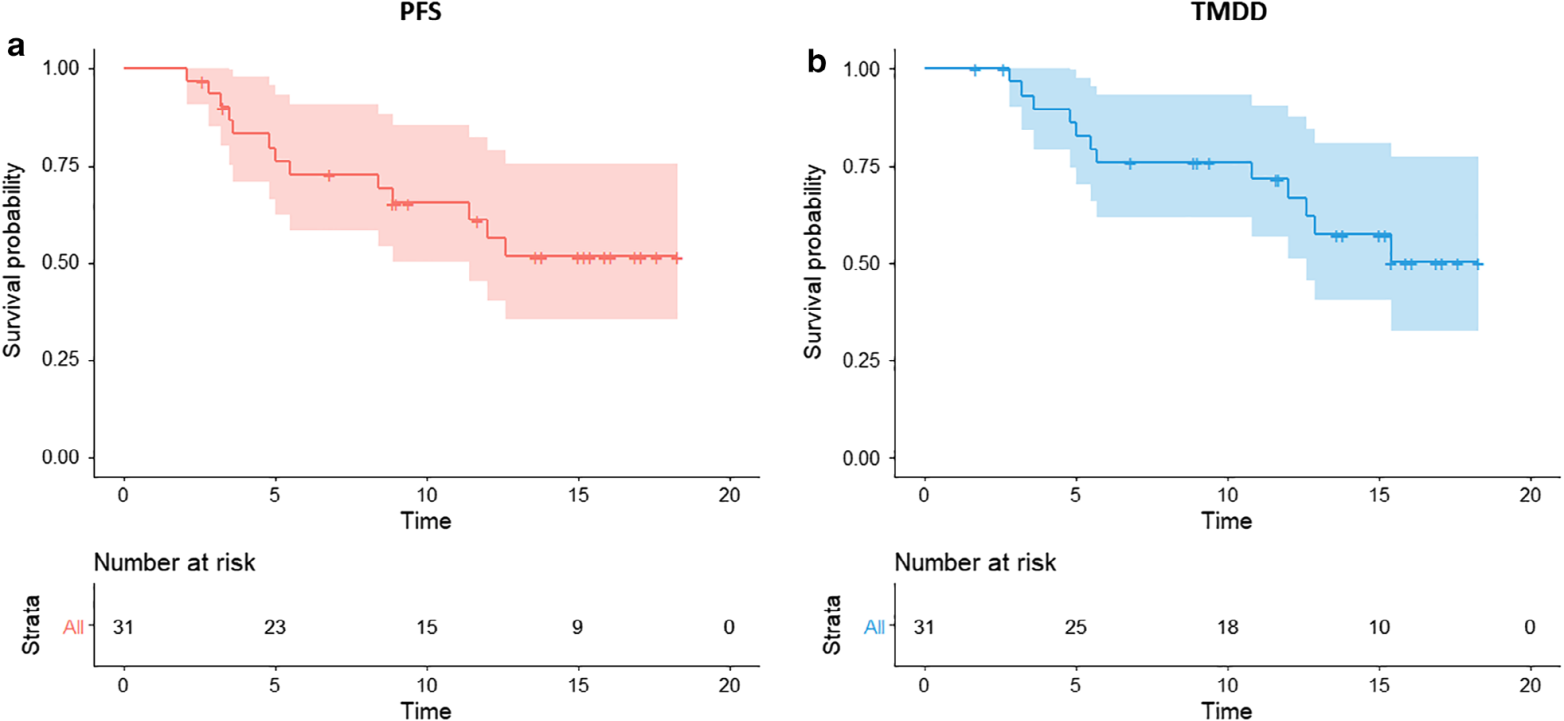
Overall efficacy analysis of the durvalumab intent‐to‐treat cohort showing the Kaplan‐Meier curve with 95% confidence interval (shaded area) of (**a**) post‐CRT PFS Strata (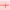) All and (**b**) post‐CRT TMDD Strata (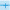) All.

The outcome of post‐CRT tumor control applying durvalumab consolidation, in terms of the post‐CRT PFS and TMDD, were analyzed for the durvalumab intent‐to‐treat cohort. At the time of analysis, Kaplan‐Meier curve showed that the median post‐CRT PFS and TMDD were both not reached whereas the 12 month PFS and TMDD‐free rate were 56.4% (Fig 2a) and 66.9% (Fig 2b), respectively. The objective responses of the intent‐to‐treat cohort, in terms of CR, PR, SD, and PD, were 0, 25.8%, 54.8%, and 12.9%, respectively and two (6.5%) patients were not assessed for the response due to not receiving durvalumab treatment (Table 2).

**Table 2:** Objective response of the study patients

Variables, n (%)	Intent‐to‐treat cohort (n = 31)
Responder	
No. of patients	8
% (95% CI)	25.8 (11.9–44.6)
Complete response‐ no. (%)	0
Partial response‐no. (%)	8 (25.8)
12‐month PFS (%)	56.4
Stable disease‐no. (%)	17 (54.8)
Progression disease‐no. (%)	4 (12.9)
Not assessed†‐no. (%)	2 (6.5)

† Not receiving study treatment.

### Association between neutrophil‐to‐lymphocyte ratio and post‐CRT PFS and TMDD

**Figure 3:**
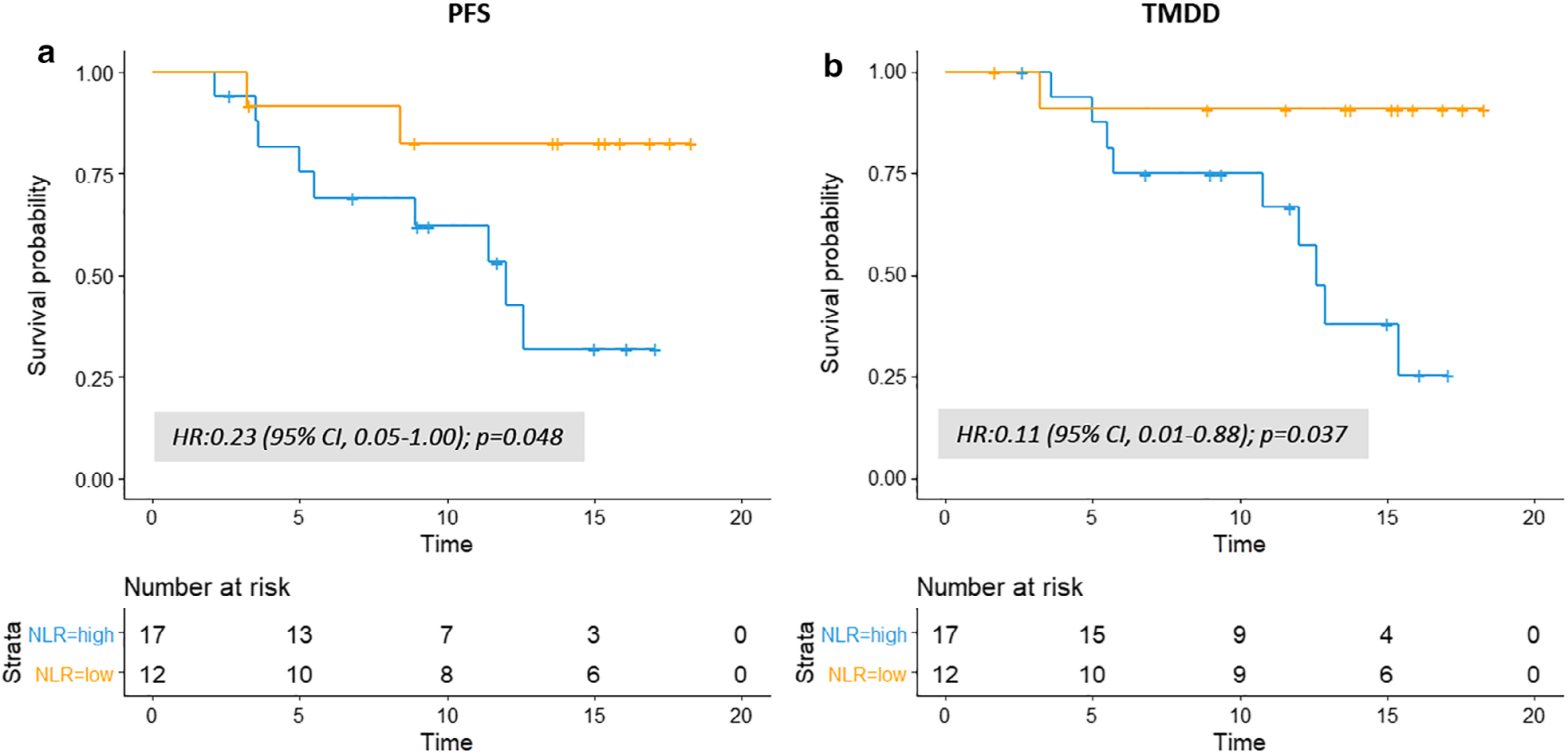
Efficacy between the high and low NLR groups of the durvalumab on‐treatment patients for (**a**) post‐CRT PFS Strata (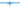) NLR = high, and (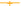) NLR = low and (**b**) post‐CRT TMDD analysis. NLR, neutrophil‐to‐lymphocyte ratio Strata (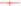) NLR = high, and (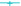) NLR = low.

**Figure 4:**
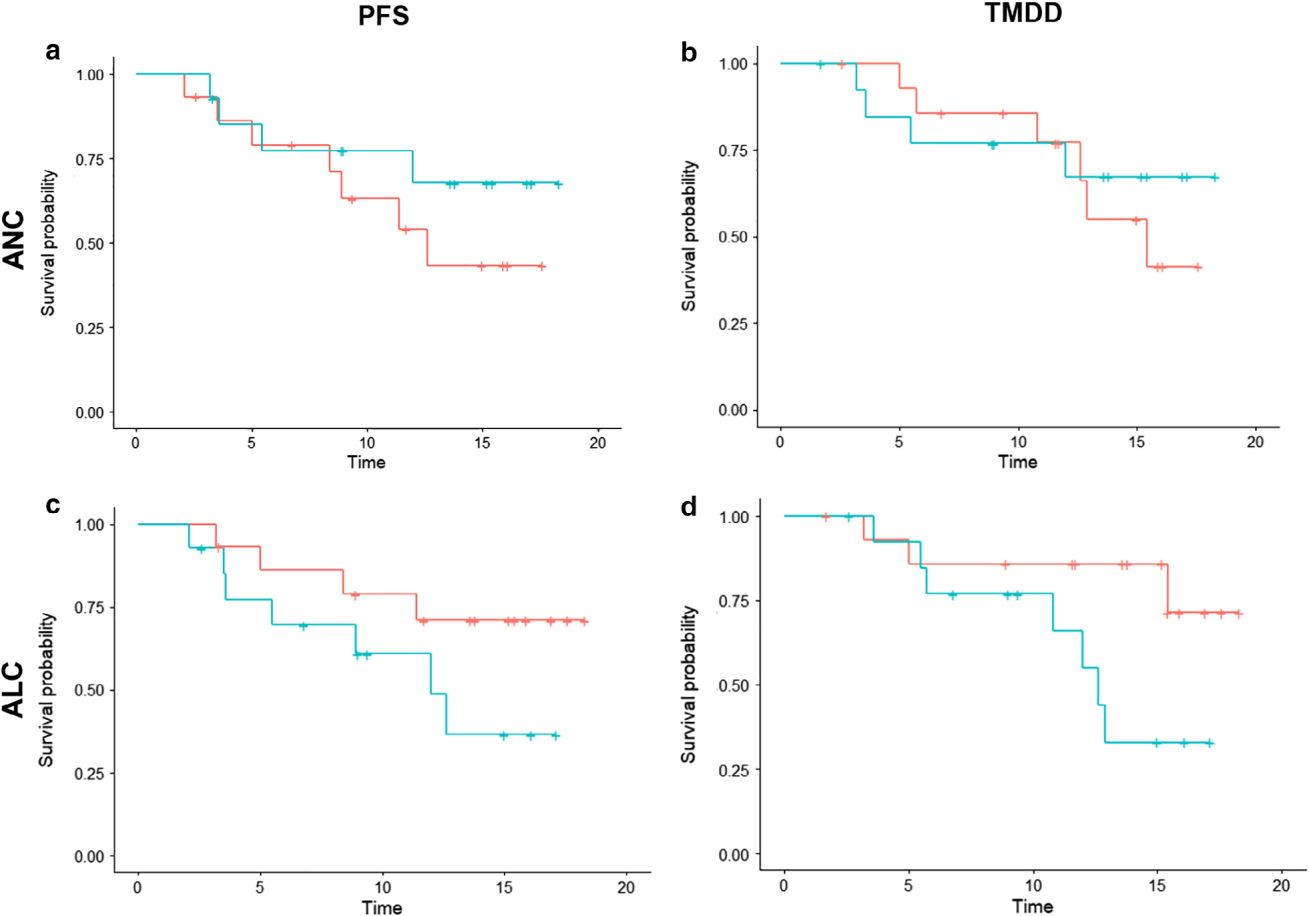
Efficacy of the durvalumab on‐treatment patients for (**a**) post‐CRT PFS Strata (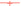) ANC = high, (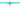) ANC = low and (**b**) post‐CRT TMDD analysis between the high and low ANC groups Strata (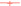) ANC = high, (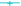) ANC = low; and efficacy of the durvalumab on‐treatment patients for (**c**) post‐CRT PFS Strata (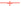) ANC = high, (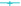) ANC = low and (d) post‐CRT TMDD analysis between the high and low ALC groups Strata (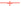) ANC = high, (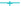) ANC = low. ANC, absolute neutrophil count; ALC, absolute lymphocyte count.

To assess the association between the peripheral blood neutrophil‐to‐lymphocyte ratio (NLR) and the post‐CRT PFS and TMDD on consolidation treatment, the patients on‐treatment with durvalumab (Fig 1) were divided based on the level of NLR, herein the NLR at 3.8, as determined by the Cutoff Finder. Most of the baseline clinical characteristics including age, sex, ECOG PS, histology, *EGFR* and *ALK* mutation status, PD‐L1 tumor proportion score, CRT protocol and timing of durvalumab initiation were similar between the high and low NLR groups (Table 3). Kaplan‐Meier curve analysis showed that compared to the high NLR group the median post‐CRT PFS of the low NLR group was significantly longer (not reach vs. 12.0 months [95% CI: 5.5–not estimable]; log‐rank test *P* = 0.040; the hazard ratio for disease progression or death, 0.23 [95% CI: 0.05–1.00]; *P* = 0.048; Fig 3a) and the 12‐month post‐CRT PFS rate was higher (82.5 vs. 42.6%; Fig 3a). In terms of the post‐CRT TMDD: low NLR group also showed a significantly longer median post‐CRT TMDD (not reach vs. 12.6 months, [95% CI: 10.8–not estimable]; log‐rank test *P* = 0.010; the hazard ratio for distant metastasis or death, 0.11 [95% CI: 0.01–0.88]; *P* = 0.037; Fig 3b) and a higher 12 month post‐CRT TMDD‐free rate (90.9 vs. 57.1%; Fig 3b) than the high NLR group. The association between the post‐CRT tumor control and the individual cell count, the absolute neutrophil count (ANC) and absolute lymphocyte count (ALC) was also assessed. A similar hazard of the post‐CRT progression or death (1.90 [95% CI: 0.55–6.52]; *P* = 0.306; Fig 4a) and of the post‐CRT distant metastasis or death (1.38 [95% CI: 0.39–4.91]; *P* = 0.617; Fig 4b) were noted between the high versus low ANC groups. For the high versus low ALC groups, the hazard of the post‐CRT progression or death (0.41 [95% CI: 0.12–1.41]; *P* = .150; Fig 4c) and of the post‐CRT distant metastasis or death (0.29 [95% CI: 0.07–1.15]; *P* = 0.070; Fig 4d) were only numerically lower.

**Table 3:** Clinical characteristics of the high and low ALC group

Variable, n (%)	high NLR (n = 17)	low NLR (n = 12)	p‐value
Age, median (range), year	63 (50–72)	63 (55–76)	0.298
Gender (male)			
Male	14 (82.4)	10 (83.3)	1.000
Female	3 (17.6)	2 (16.7)	
Smoking status			
Smoker/ex‐smoker	13 (76.5)	8 (66.7)	0.873
Never smoker	4 (23.5)	4 (33.3)	
ECOG PS			
0	14 (82.4)	11 (91.7)	0.652
1	2 (11.8)	1 (8.3)	
2	1 (5.8)	0	
Histology			
Adenocarcinoma	10 (58.8)	10 (83.3)	0.318
Non‐adenocarcinoma	7 (41.2)	2 (16.7)	
Staging			
IIIA	3 (17.6)	5 (41.7)	0.319
IIIB	13 (76.5)	6 (50.0)	
IIIC	1 (5.9)	1 (8.3)	
*EGFR* mutation status			
Mutated *EGFR*	2 (11.8)	2 (16.7)	1.000
Wild type	10 (58.8)	8 (66.6)	
Unknown	5 (29.4)	2 (16.7)	
*ALK* fusion status			
Positive	1 (5.9)	0	0.556
Negative	10 (58.8)	6 (50.0)	
Unknown	6 (35.3)	6 (50.0)	
PD‐L1 TPS			
Positive (≥1%)	10 (58.8)	4 (33.3)	0.278
Negative (<1%)	2 (11.8)	4 (33.3)	
Unknown	5 (29.4)	4 (33.3)	
Chemotherapy regimen			
Docetaxel plus cisplatin	6 (35.3)	4 (33.3)	1.000
Vinorelbine plus cisplatin	11 (64.7)	8 (66.7)	
Dose of radiotherapy			
60–66 Gy	13 (76.5)	10 (83.3)	1.000
>66 Gy	4 (23.5)	2 (16.7)	
Timing of durvalumab initiation post‐CCRT, median (month)	2.6 (1.2–3.5)	3.2 (2.5–3.8)	0.318

ALC, absolute lymphocyte count; ECOG PS, Eastern Cooperative Oncology Group performance status; EGFR, epidermal growth factor receptor; ALK, anaplastic lymphoma kinase; PD‐L1, Programmed death‐ligand 1; TPS, tumor proportion score; CCRT, concurrent chemoradiation.

### Adverse events profile of durvalumab treatment

The most commonly noted all grade adverse events in patients receiving post‐CRT durvalumab consolidation included skin rash (seven patients; 24.1%), pruritus (five patients; 17.2%), pneumonitis (five patients; 17.2%), elevated AST/ALT (three patients; 10.3%), diarrhea (three patients; 10.3%) and cough (three patients; 10.3%; Table 4). Overall serious adverse events of grade 3–5 were noted in four (13.8%) patients in which pneumonitis were noted in two (6.9%) patients, skin rash in one (3.4%) patient and elevated AST/ALT in one (3.4%) patient. Logistic regression was performed (Table 5) to analyze the clinical factors predictive of pneumonitis, including age, ECOG PS, smoking status, chemotherapeutic agents used, PD‐L1 status and NLR. However, none of them were found to be associated with the development of pneumonitis in the analysis.

**Table 4:** Treatment‐related adverse events

	Durvalumab (n = 29)	
Frequency n (%)	Any grade	Grade ≥ 3
Any event	21 (72.4)	4 (13.8)
Skin rash	7 (24.1)	1 (3.4)
Pruritus	5 (17.2)	0
Nausea/Poor appetite	1 (3.4)	0
Diarrhea	3 (10.3)	0
Elevated AST or ALT	3 (10.3)	1 (3.4)
Elevated amylase or lipase	2 (6.9)	0
Constipation	1 (3.4)	0
Pneumonitis	5 (17.2)	2 (6.9)
Cough	3 (10.3)	0
Sore throat	1 (3.4)	0
Headache	1 (3.4)	0

**Table 5:** Regression analysis of the factors associated with pneumonitis

Variables	Odd ratio (95% C.I.)	*P*‐value
Age (≥65 *vs*. <65)	2.10 (0.29–15.0)	0.454
ECOG PS (0 *vs*. ≥ 1)	0.57 (0.05–6.98)	0.661
Smoking status (smoker/ex‐smoker *vs*. non‐smoker)	1.65 (0.16–17.5)	0.679
Chemotherapy agent (docetaxel *vs*. vinorelbine)	0.75 (0.10–6.57)	0.776
PD‐L1 TPS (positive *vs*. negative)	0.33 (0.03–3.20)	0.341
NLR (low *vs*. high)	2.50 (0.35–21.9)	0.362

## Discussion

This work reported the preliminary results of the post‐CRT PFS and TMDD with durvalumab consolidation treatment in an intent‐to‐treat cohort of stage III unresectable NSCLC patients who participated the durvalumab EAP in a real‐world setting. We noted a 56.4% post‐CRT 12 month PFS rate and a 66.9% post‐CRT 12 month TMDD‐free rate in this real‐world intent‐to‐treat cohort. When the NLR level, but not those of the ANC or ALC, was taken into account at the administration of durvalumab, we noted a significantly longer post‐CRT PFS and TMDD for the low NLR patients in the durvalumab on‐treatment cohort. The all grade adverse events were noted in 72.4% of the on‐treatment patients in which pneumonitis was found in 17.2%. However, clinical factors did not show predictive effect to the development of pneumonitis in this analysis.

The consolidation treatment for the stage III unresectable NSCLC patients, usually given through chemotherapeutic agents, has been a controversial practice mainly due to the tolerability secondary to the side effects after the completion of concurrent CRT. In this analysis, the median time between the completion of concurrent CRT and the initiation of durvalumab consolidation was 2.8 months. This time window, somewhat longer than that in the reference PACIFIC study, can be partly explained by the patients' post‐CRT tolerability as well as the physician's precaution toward consolidation treatment immediately after the completion of CRT in a real‐world setting. Nevertheless, as patients may experience disease progression in this window, the influence was taken into account by applying the intent‐to‐treat definition to the EAP cohort. With this definition, two (6.5%) patients who had disease progression in the window and had not received durvalumab were included in the analysis to avoid the overestimation of the effect of durvalumab treatment. Following this principle, we demonstrated the post‐CRT 12‐month PFS rate and the TMDD‐free rate in this real‐world cohort were similar to those in the PACIFIC trial. On the other hand, the 25.8% response rate of the durvalumab consolidation in this analysis, similar to that 30.0% in the PACIFIC trial, was also noted.

In addition, the present study showed the association between the level of NLR and the post‐CRT tumor control with durvalumab treatment. Previous studies of advanced melanoma patients receiving immune checkpoint inhibitors have shown that peripheral blood makers may play a prognostic as well as a predictive role in their treatment. Giacomo *et al*.,^23^ and Delyon *et al*.,^24^ reported that a high lymphocyte count, or a change in the count slope at the start of ipilimumab treatment, were correlated with better overall survival (OS) and the treatment response; a finding which was similarly noted in a Japanese melanoma cohort receiving ipilimumab treatment.^25^ On the other hand, the significance of the neutrophil count was also highlighted by the work of Capone *et al*. in which the high neutrophil‐to‐lymphocyte ratio was correlated with a worse OS and PFS.^26^ Recently, in advanced NSCLC patients treated with PD‐1/PD‐L1 inhibitors, the derived neutrophil‐to‐lymphocyte ratio was shown to be associated with the OS and PFS^19^ whereas this association was not observed in patients who received chemotherapy. In this preliminary report involving locally advanced NSCLC patients who received post‐CRT durvalumab treatment, the correlation between the baseline NLR and the post‐CRT disease progression and distant metastasis was first demonstrated. The level of the individual type of white blood cells, in terms of the ANC and the ALC, showed less significant clinical implication in this analysis.

In this real‐world EAP cohort, we noted a 17.2% of patients had treatment‐related pneumonitis in which 6.9% required a discontinuation or interruption of durvalumab. A number of clinical factors, including the histology of squamous cell carcinoma and poorer ECOG PS at 1, were noted to be associated with the development of pneumonits in the PACIFIC study whereas no specific clinical factors predictive of the development of pneumonits were identified in this analysis. In addition, 6.9% of the patients underwent treatment‐related elevation of amylase and lipase, which was a finding not commonly reported elsewhere. However, this treatment‐related abnormality was usually mild and resolved spontaneously.

The major limitation of this study was the small sample size. However, the difference between the high and low NLR groups in terms of the post‐CRT PFS and TMDD with durvalumab treatment remained statistically significant at this sample size, suggesting the significance of NLR in relation to the efficacy of durvalumab treatment and thereby warrants further investigation in the future. In addition, the retrospective nature of this analysis may have underreported the toxicity profiles as well as the grading, particularly those that were non‐severe and should therefore be interpreted with caution. In conclusion, durvalumab consolidation showed substantial efficacy in real‐world locally advanced unresectable NSCLC patients who underwent concurrent CRT, and the level of NLR at the initiation of durvalumab was associated with the treatment efficacy.

## Disclosure

All of the authors have no conflict of interest to disclose.
